# Role of Efflux Pumps and Intracellular Thiols in Natural Antimony Resistant Isolates of *Leishmania donovani*

**DOI:** 10.1371/journal.pone.0074862

**Published:** 2013-09-17

**Authors:** Smita Rai, Bhaskar, Sudhir K. Goel, Upendra Nath Dwivedi, Shyam Sundar, Neena Goyal

**Affiliations:** 1 Division of Biochemistry, CSIR-Central Drug Research Institute, Lucknow, India; 2 Department of Biochemistry, All India Institute of Medical Sciences, Bhopal, India; 3 Department of Biochemistry, University of Lucknow, Lucknow, India; 4 Institute of Medical Sciences, Banaras Hindu University, Varanasi, India; Technion-Israel Institute of Technology, Haifa, Israel

## Abstract

**Background:**

In view of the recent upsurge in the phenomenon of therapeutic failure, drug resistance in *Leishmania* , developed under natural field conditions, has become a great concern yet little understood. Accordingly, the study of determinants of antimony resistance is urgently warranted. Efflux transporters have been reported in *Leishmania* but their role in clinical resistance is still unknown. The present study was designed to elucidate the mechanism of natural antimony resistance in *L. donovani* field isolates by analyzing the functionality of efflux pump(s) and expression profiles of known genes involved in transport and thiol based redox metabolism

**Methodology/Principal Findings:**

We selected 7 clinical isolates (2 sensitive and 5 resistant) in addition to laboratory sensitive reference and SbIII resistant mutant strains for the present study. Functional characterization using flow cytometry identified efflux pumps that transported substrates of both P-gp and MRPA and were inhibited by the calmodulin antagonist trifluoperazine. For the first time, verapamil sensitive efflux pumps for rhodamine 123 were observed in *L. donovani* that were differentially active in resistant isolates. RT-PCR confirmed the over-expression of MRPA in isolates with high resistance index only. Resistant isolates also exhibited consistent down regulation of AQP1 and elevated intracellular thiol levels which were accompanied with increased expression of ODC and TR genes. Interestingly, γ-GCS is not implicated in clinical resistance in *L. donovani* isolates.

**Conclusions/Significance:**

Here we demonstrate for the first time, the role of P-gp type plasma membrane efflux transporter(s) in antimony resistance in *L. donovani* field isolates. Further, decreased levels of AQP1 and elevated thiols levels have emerged as biomarkers for clinical resistance.

## Introduction

Leishmaniasis comprises a complex of vector-borne diseases, caused by more than 20 species of the protozoan genus *Leishmania* , that range from localized skin ulcers to lethal systemic disease [1]. Leishmaniasis is classified as one of the ‘‘most neglected diseases’’ [2], based on the limited resources invested in diagnosis, treatment, and control, and its strong association with poverty [3]. Since, there are no vaccines against Leishmaniasis available at present [4], chemotherapy is the main control strategy and pentavalent antimonials (SbV) remain the mainstay. However, the efficacy of SbV is now threatened by the emergence of drug resistant *Leishmania* parasites, as described in several endemic regions [5-9]. Among alternative drugs, pentamidine is toxic with reported cases of resistance; amphotericin B is both expensive and toxic [10] and oral miltefosine is limited by cost, contraindications, and emerging relapse [11,12]. Therefore, resistance to first line drug(s) has a very big impact on the treatment of Leishmaniasis. The present-day requirement in the treatment of Leishmaniasis is to battle escalating antimony unresponsiveness and hence an urgent need exists to define the mechanisms of resistance in field.

The mechanisms of resistance to antimony in *Leishmania* have largely emerged from studies conducted on laboratory-generated drug-resistant cell lines generated through step wise exposure to either antimony or related metal arsenic [13]. A consistent resistance mechanism deduced from *in vitro* studies involves reduced accumulation of active drug, trivalent antimony (SbIII) in parasite either due down regulation of uptake transporter, aquaglyceroporin (AQP1) [14], or increased sequestration of drug-thiol conjugate in vacuole due to up regulation of ABC transporter, P-glycoprotein A (P-gpA) also named as multi drug resistant related protein A (MRPA) in parasite [15,16], accompanied with elevated levels of unique parasite thiol, trypanothione and over-expression of its biosynthetic pathway enzymes [17,18]. Over the last few years, the focus has been shifted towards exploring the mechanisms of antimony resistance in clinical isolates. Interestingly, a common mechanism of drug resistance was not observed operating in the isolates of either same species from different countries and in different species from same country. For example, AQP1 transcript levels exhibited consistent down regulation in the field isolates of *Leishmania donovani* from India [19] and Nepal [20,21] but RNA levels remain unaltered in resistant isolates of *L. braziliensis* and *L. guyanensis* [22,23]. Similarly, gene amplification accompanied with up-regulation of MRPA gene was observed in *L. donovani* isolates only from India [24,25] but neither from Nepal nor in *L. braziliensis* and *L. guyanensis* [22,23]. Moreover, the studies to ascertain the functionality of this transporter protein in clinical resistance remained inconclusive. In addition, increased intracellular thiol levels [24], specifically glutathione and cysteine had been implicated in clinical resistance but the levels of trypanothione remained unaltered [25]. The precursor protein for glutathione biosynthesis, γ-GCS was neither amplified and nor up-regulated in *L. donovani* Indian resistant isolates [24,25] but down regulation of γ-GCS was observed in Nepalese isolates [20,21]. In *L. guyanensis*, γ-GCS was over expressed in therapeutic failure isolates [22]. Similarly, the precursor protein of spermidine biosynthesis, ODC was amplified at the genetic and protein levels in Indian *L. donovani* resistant isolates [25] and in *L. braziliensis* [23], but the gene was down regulated in isolates from Nepal [20]. So far, to characterize resistance mechanism in Indian *L. donovani* isolates, limited parameters had been studied in a small number (1-3) of isolates [19-21,25,26] unlike *L. braziliensis* or *L. guyanensis*, where much larger number of isolates was evaluated. Hence the conducted studies failed to provide a defined mechanism of resistance operating in field conditions. Therefore, more comprehensive studies are required to resolve this ambiguity.

In the present study we have tried to elucidate the mechanism of natural antimony resistance in *L. donovani* isolates, isolated from VL patients in Bihar/eastern UP, India, by analyzing the expression profiles of known genes involved in transport and thiol based redox metabolism followed by characterization of the functionality of efflux pump(s) and related enzymes. For the first time, a parallel comparison was made with a laboratory raised *L. donovani* mutant strain resistant to 450 µM SbIII.

## Materials and Methods

### Materials

Standard biochemical reagents, SbIII (potassium antimony tartarate hydrate) and Amphotericin B were obtained from Sigma. Medium 199, fetal bovine serum and Superscript II RNase H-Reverse Transcriptase were from Invitrogen. iQ Sybr Green Supermix was procured from Bio-Rad. Biomol green reagent was from Enzo Life Sciences.

### Ethics statement

The ethics committee of the Kala-azar Medical Research Center (Muzzaffarpur, India) reviewed and approved the study protocol. Written informed consent was obtained from every subject enrolled into the study. Institutional Animal Ethics Committee (IAEC) of CSIR-Central Drug Research Institute, Lucknow, reviewed and approved the animal protocol (87/10/Biochem/IAEC/Renew02(90/11) which was adhered to National guidelines CPCSEA (Committee For the Purpose of Control and Supervision of Experiments on Animals) of Government of India. Animals were housed in plastic cages in climatically controlled rooms and fed with standard rodent food pellet (Lipton India, Bombay) and water ad libitum.

### Clinical isolates

The clinical strains of *L. donovani* were isolated from patients of Kala-azar Medical Center of the Institute of Medical Sciences, Banaras Hindu University (Varanasi, India) and from its affiliated hospital at Muzzafarpur, Bihar. The criteria of diagnosis of visceral Leishmaniasis were the presence of Leishman Donovan bodies in splenic aspirates, which were graded according to standard criteria [27]. After diagnosis, the patients were administered intravenously one course of SSG (20 mg/kg of body weight/day for 30 days). Response to treatment was evaluated by repeating splenic aspiration at day 30 of treatment. The designation of patients was based on the absence of fever, clinical improvement with reduction in spleen size and the absence of parasites in aspirates. Patients who had parasites were considered to be unresponsive to antimony. These patients were subsequently treated with amphotericin B. Some patients, belonging to resistant area were treated directly with amphotericin B. Cryopreserved parasites were used for experimental work within six passages after isolation from the patients and were maintained in absence of drug pressure *in vitro* during the experiments.

### Reference strain

*L. donovani* promastigotes, Dd8 strain (World Health Organization designation MHOM/IN/80/Dd8), which was originally obtained from (late) Prof. P.C.C. Garnham (Imperial College, London, United Kingdom), was used as the sensitive reference strain. It was maintained at CSIR-Central Drug Research Institute in golden hamsters.

### Selection of laboratory SbIII resistant mutant

*L. donovani* Dd8 promastigotes resistant to trivalent salt of antimony, were selected by gradual increases in the concentration of compound (potassium antimony tartrate, SbIII) until the cells were able to grow normally at 450 µM concentration. Resistant mutant cells (Mt) were then maintained under continuous drug pressure.

### Culture conditions

The splenic aspirates of patients were inoculated into NNN medium, grown at 25°C, and sub-cultured every sixth day. The positive cultures were then adapted to medium 199 (Sigma) supplemented with 10% fetal calf serum and 1% penicillin (50 U/ml) and streptomycin (50 µg/ml) solution (Sigma) for mass cultivation.

### *In vitro* SbIII susceptibility of clinical isolates

The trivalent antimony compounds are presumed be the active form of the drug because they are highly active against both promastigote and amastigote stages of the parasite. The 50% inhibitory concentration (IC_50_) of SbIII was determined as an index of antimony resistance phenotype of the isolates under laboratory conditions. Exponentially growing parasites were seeded in 96-well microplates (0.2× 10^6^/well) in medium 199 supplemented with 10% FBS. Cells were allowed to grow in presence or absence of drug(s) for 48 hours at 24 ± 1°C. The number of viable cells per well was determined microscopically and the IC_50_ value was calculated by probate analysis. Amphotericin B was used as the reference drug.

### RNA isolation and real-time PCR

Total RNA was isolated from 1x10^7^ promastigotes of mid-log phase using the TRIzol reagent (Invitrogen) as described by the manufacturer. The RNAs were treated with RNase-free DNase I (Fermentas) to avoid any genomic contamination and further purified using RNeasy columns (Qiagen). Complementary DNA was re-synthesised from 5µg of total RNA using Super ScriptTM II RNase H-Reverse Transcriptase and random hexamer primer. Real time PCR was performed for expression profiling of five genes involved in influx, sequestration of antimony, thiol metabolism of parasite using iQ Sybr Green Supermix and forward and reverse primers as specified in Table 1. Alpha tubulin gene was included for normalization purposes, referred to as internal control. Reactions were run on a LightCycler (Roche). The PCR was immediately followed by a melt curve analysis using temperature increments of 0.5°C every 30 s to ascertain if the expected product was amplified and to ensure no nonspecific products or primer dimers (which could bias the quantification) were formed. All reactions were done in triplicate. The relative amount of PCR products generated from each primer set was determined based on threshold cycle (Ct) value of the gene of interest, normalised to that of reference α-tubulin gene using Livak method [28].

**Table 1:** Chosen internal control and target genes; primer design and PCR conditions.

**Gene**	**Protein**	**Sequence of forward and reverse primers**	**Final primer conc.(nM)**	**Annealing temperature (°C)**
ATUB	Alpha-tubulin	5’AGGATGCGGCGAACAACTAC3’	300	61
		5’CAGCGTGGAACACCATAAAGC3’		
MRPA	ABC transporter	5’CGCAGCCGTTTGTGCTTGTGG3’	300	55
		5’ TGCCGTACGTCGCGATGGTGC3’		
AQP1	Aquaglyceroporin1	5’ TGGCGAACTGTGTCTTTGGT3’	500	46
		5’GTACTTGGACGCCATCGT3’		
γ-GCS	γ-Glutamylcysteine synthetase	5’ACATTGGCTGGCGCGTTGAGT3’	500	46
		5’GACGATGGAGATCTTGGTGTA3’		
ODC	Ornithine decarboxylase	5’ CAGCGGCAACGACGACCAGT3’	500	61
		5’GTGACATCACCGCCGCCGAA3’		
TR	Trypanothione reductase	5’ GGCGAGGTTCTGGGTGTTC3’	300	58
		5’GACTCCGATGGTGCTGTGG3’		

### Flow cytometric analysis

Monitoring of dye accumulation and retention was carried out on a flow cytometer (FACS Calibur, Becton Dickinson) equipped with an argon-ion laser (15 MW) tuned to 488 nm. Data analysis was carried out with Cell Quest (BD) software. Fluorescence of rhodamine 123 and calcein was measured in the photomultiplier tube designated FL1, which is equipped with a 530/30-nm band pass filter. Samples were analyzed at the flow rate of 100–200 cells/sec and a typical analysis was based on examination of 10,000 cells. Accumulation of rhodamine 123 (Rho) was studied by incubating the promastigotes with 1 µg/ml Rho in medium 199 at 24 ± 1°C for 1 h in the presence or absence of inhibitors, 100 µM verapamil and 20 µM trifluoperazine. After incubation, the cells were washed with cold PBS and then subjected to FACS analysis. Calcein uptake was studied by incubating parasites with 1 µM calcein-AM at 24 ± 1°C for 1 h 30 min in the presence or absence of inhibitors, 20 µM probenecid and 20 µM trifluoperazine. Sodium azide treatment was given by incubating the parasites with NaN_3_ for 15 min at 24 ± 1°C prior to loading. Efflux of dyes was studied after washing the loaded parasites twice with chilled PBS pH 7.4 and re-suspended in plain medium M199 either in presence or absence of inhibitor(s).

### Preparation of plasma membrane vesicles

Everted plasma membrane vesicles were prepared as described previously [29] with some modifications. Briefly, late log phase parasites were harvested and washed thrice with ice-cold PBS, pH 7.2. The pellet was suspended in hypotonic lysis buffer (1 mM Tris-Cl pH 7.9, 1 mM EDTA, 0.5 mM PMSF, 8 µg/ml aprotinin, 10 µg/ml leupeptin) and the cells were disrupted by sonication three times with the pulse setting of 30 s followed by 30 s time interval. 200 µl TMEP buffer (50 mM Tris-Cl pH 7.0, 50 mM mannitol, 2 mM EGTA, 0.5 mM PMSF, 8 µg/ml aprotinin, 10 µg/ml leupeptin, 2 mM β-mercaptoethanol) was added per ml of lysate. Undisrupted cells and nuclear debris were removed by centrifugation at 10,000 x g for 1 min. The supernatant was then diluted with 2 volumes of TMEP buffer and centrifuged at 17,000 x g for 40 min to remove organelles and other intracellular membranes. The supernatant was collected and further centrifuged at 140,000 x g for 30 min. The pellet was suspended in TMEP buffer and stored at -80°C till further use. This preparation has been reported to be highly enriched in plasma membrane vesicles [30,31].

### ATPase activities of plasma membrane vesicles

The ATPase activities of parasite plasma membrane vesicles were determined by measuring inorganic phosphate liberation [29]. The standard assay mixture (0.1 ml final volume) contained 50 mM Tris-Mes buffer (pH 6.8), 2 mM EDTA, 2 mM ouabain, 2 mM DTT, 50 mM KCl, 5 mM sodium azide and 20 µg protein of the plasma membrane fraction. The reaction was started with the addition of 5 mM MgATP and allowed to proceed for 20 min at 37°C. The reaction was stopped by addition of 1 ml Biomol, Green reagent and after 30 min incubation, the amount of released inorganic phosphate was determined by measuring optical density at 660 nm. The ATPase activity was calculated after subtracting the non-specific ATP hydrolysis measured in the absence of plasma membranes taking inorganic phosphate as standard.

### Analysis of thiols

Total intracellular thiols in promastigotes were estimated in de-proteinized cell extracts [32]. Briefly, cells at mid log phase (3 × 10^7^/ml) were harvested, washed with PBS (pH 7.4), suspended in an equal volume of 10% trichloroacetic acid. The cell suspension was freezed and thawed once and centrifuged at 10,000 × *g* for 10 minutes at 4°C. The thiol content of the supernatant was determined with 0.6 mM DTNB in 0.1 M sodium phosphate buffer (pH 8.0). The developed yellow color was measured at 412 nm. The reduced glutathione was taken as the standard and total cell thiols were represented as total glutathione.

L-buthionine-(SR)-sulfoximine (BSO) was added to the promastigote suspension (S1, R5, Dd8 and Mt) at a concentration of 5 mM for 48 hours. After BSO treatment, cells were resuspended in fresh medium (without BSO), supplemented with 10% FCS and incubated for 3h at 24°C to regenerate the depleted thiols. Total intracellular thiols before and after BSO treatment and regeneration were measured using CMFDA as probe by flowcytometry [33].

### Gamma GCS activity

Late-log phase *Leishmania* cells were pelleted and re-suspended in 5 mM Tris–HCl pH 8.0. Cells were disrupted by sonication (Sonics) twice with pulse setting of 10 s with time interval of 20 s. The supernatant was freed of particulate material by centrifugation (14,972 x g for 40 min) followed by ultracentrifugation (130,000 x g for 60 min) and used as source of enzyme. The γ-GCS activity was determined following the formation of ADP in coupled assay with pyruvate kinase and lactate dehydrogenase [34]. The reaction mixture (final volume, 1.0 ml) contained Tris–HCl buffer (100 mM, pH 8.2), sodium L-glutamate (10 mM), L-cysteine (10 mM), magnesium chloride (20 mM), disodium ATP (5 mM), sodium phosphoenolpyruvate (2 mM), potassium chloride (150 mM), NADH (0.2 mM), pyruvate kinase (10 U) and lactate dehydrogenase (10 U). The reaction was initiated by addition of the cell supernatant, and the rate of decrease in absorbance at 340 nm was followed at 25°C. One unit of enzyme activity is defined as the amount that catalyzes the formation of 1 mmole of ADP per hour. Specific activity is expressed as units/milligram of protein

### Activity of trypanothione reductase

Trypanothione reductase activity in crude cell extracts of both sensitive and resistant strains was assayed spectrophotometrically at 412 nm, as previously described [35].

## Results

### Characterization of field isolates

The clinical and laboratory profiles of VL patient isolates are summarized in Table 2. Clinical isolates obtained from VL patients who had responded to SAG chemotherapy were designated as SAG-sensitive, whereas VL patients who did not respond to SAG were designated as SAG-resistant. Others were graded initially on the basis of their area of collection like resistant or sensitive area and finally on their response to antimony (SbIII) under *in vitro* conditions. All the clinical isolates except S1 and R5, exhibited corresponding SbIII resistance phenotype under laboratory conditions (Table 2). The isolate S1 was collected from resistant area, Muzzafarpur but exhibited antimony sensitivity with resistance index of 0.712 hence was designated as sensitive isolate. Similarly, R5 was collected from sensitive area but exhibited significant resistance to SbIII with resistance index of 3.18 hence was designated as resistant isolate. The abbreviation Mt was used for the laboratory generated mutant of *L. donovani* Dd8 strain that was resistant to 450 µM concentration of SbIII and was grown under constant drug pressure. The SbIII susceptibility of field isolates as well as laboratory mutant was drug specific with no cross resistance to the second-line line drug, Amphotericin B (data not shown).

**Table 2:** *L. donovani* isolates from India tested for their *in vitro* SbIII susceptibility and linked with clinical response.

**Isolates**	**Area**	**Drug response**	**LD score**	**IC_50_ (µM)**	**Index of SbIII resistance**
**Sensitive isolates:**					
Dd8 (MHOM/IN/80/Dd8)	NA	NA	NA	97	1
158-S1	Muzaffarpur	Resistant area	1+	69	0.712
155-S	Pard 111/3	Sensitive area	2+	84	0.867
**Resistant isolates:**					
151-R1	Muzaffarpur	Resistant SAG	NA	187	1.93
90-R2	Muzaffarpur	Resistant area	2+	194	2.2
77-R3	Muzaffarpur	Resistant SAG	1+	227	2.34
144-R4	Muzaffarpur	Resistant	NA	307	3.17
		amphotericin B			
93-R5	Ballia (BHU/NMW-17)	NA	4+	308	3.18
**Lab raised mutant:**					
Mt*	NA	NA	NA	>450	> 4.64

* Mt stands for laboratory raised mutant of *L. donovani* resistant to 450 µM potassium antimonyl tartarate hydrate (SbIII).

### Modulation of trancript levels of genes putatively involved in drug transport

#### AQP1 expression is down regulated in resistant isolates

**Figure 1:**
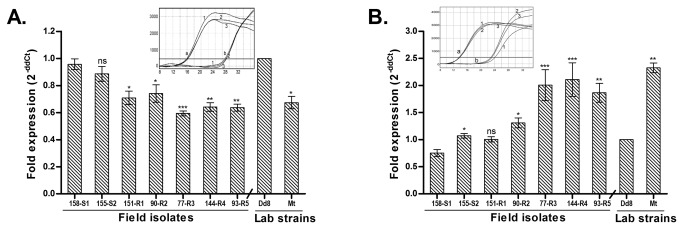
Real time PCR expression analysis of genes putatively involved in drug transport across membranes in *L. donovani* isolates. Expression ratios of resistant isolates (R1-R5) were relative to sensitive isolate S1 while laboratory resistant mutant Mt was compared with laboratory sensitive Dd8 strain. A. AQP1; B: MRPA. Results are mean of three independent experiments performed from three different RNA preparations. * P ≤ 0.05, ** P ≤ 0.005, *** P ≤ 0.0005 indicate statistical significance with respect to reference sensitive strains S1 and Dd8 respectively; ns indicates no statistically significant difference. Inset shows the RT-PCR amplification curves; set a: curves for alpha tubulin amplification, set b: curves for MRPA / AQP1 (curve1: Dd8, 2: 144-R4, 3: Mt). X axis represents PCR cycle and Y-axis represents fluorescence.

All resistant isolates (R1 – R5) exhibited invariably significant down regulation of AQP1 transcript levels when compared to sensitive field isolate S1 (1.35, 1.29, 1.6, 1.49 and 1.5 fold) respectively (Figure 1A). Interestingly, laboratory resistant mutant Mt also showed 1.48 fold down regulation in AQP1 RNA levels as compared to the sensitive reference strain Dd8, which was comparable to resistant field isolates.

#### MRPA is over expressed in resistant isolates

As shown in Figure 1B, MRPA expression in sensitive isolates S1 and S2 was comparable to the reference sensitive strain Dd8. As compared to sensitive strain S1, all resistant field isolates except R1 exhibited significant up-regulation in transcript levels (1.74, 2.66, 2.81 and 2.48 folds in R2, R3, R4 and R5) respectively. Similarly, lab resistant mutant Mt also exhibited 2.3 – fold up-regulation as compared to the reference sensitive strain Dd8.

#### Resistant isolates possess increased P-ATPase activity

Everted vesicles prepared from the plasma membranes of *L. donovani* field isolates were used for the measurement of ATPase activity in the absence and presence of sodium orthovanadate, a potent inhibitor of P-ATPases. The vanadate sensitive component of the membrane ATPase formed the P-ATPase activity. The sensitive isolates S1 and S2 exhibited comparable P-ATPase activities to laboratory sensitive strain Dd8 (Table 3). All resistant isolates showed significantly increased P-ATPase activity (1.23-3.92 folds) as compared to sensitive isolate S1. Mt also exhibited 3.19 fold increased P-ATPase activity as compared to the reference sensitive strain Dd8. Increase in P-type ATPase activities in resistant isolates including Mt suggested involvement of plasma membrane drug efflux pumps in addition to sequestration.

**Table 3:** ATPase activity of plasma membrane vesicles of *L. donovani* isolates.

**Isolates**	**Membrane ATPase activity (a)**	**Membrane ATPase activity in presence of orthovanadate (b)**	**P- ATPase activity (a-b)**	**Fold change (P-ATPase activity)**
158-S1	454 ± 40.71*	265 ± 5.80	190 ± 34	1
155-S2	478 ± 20.36*	381 ± 2.91	96 ± 17	0.5
151-R1	615 ± 40.83	380 ± 45.11	234 ± 4*	1.23
90-R2	551 ± 11.65	172 ± 2.91	379 ± 9*	2
77-R3	925 ± 16.02**	409 ± 4221	486 ± 55*	2.55
144-R4	977 ± 33.52**	233 ± 17.48	744 ± 16**	3.91
93-R5	684 ± 1.95**	221 ± 14.73	462 ± 13**	2.43
**Lab strains**				
Dd8	571 ± 5.82.4	421 ± 27.32	150 ± 22	1
Mt	667 ± 52	188 ± 18.91	479 ± 71*	3.19

ATPase activity is expressed as nmol Pi x h^- 1^ x mg^- 1^. The data are expressed as mean ± SD of three experiments with different membrane preparations. (a) represents total ATPase activity, (b) represents the ATPase activity in presence of 250 µM sodium orthovanadate. *P ≤ 0.05, **P ≤ 0.005; statistically significant difference when compared resistant isolates with sensitive isolate S1 and lab mutant Mt to reference sensitive strain Dd8.

### Functional characterization of Efflux pumps

#### Dye Accumulation studies using MDR probe Rhodamine 123

Rhodamine 123 a fluorescent cationic dye accumulates in the mitochondrion and is an established substrate for P-glycoprotein (P-gp). It has been applied as a molecular probe in studies pertaining to multidrug resistant phenotypes [36,37]. Rho123 was used in the present study to investigate whether resistance phenotype in field is associated with functionality of MDR type ATP dependent efflux pump or it is only due to sequestration.

#### Verapamil blocked the efflux of Rho 123 partially and reversibly

**Figure 2:**
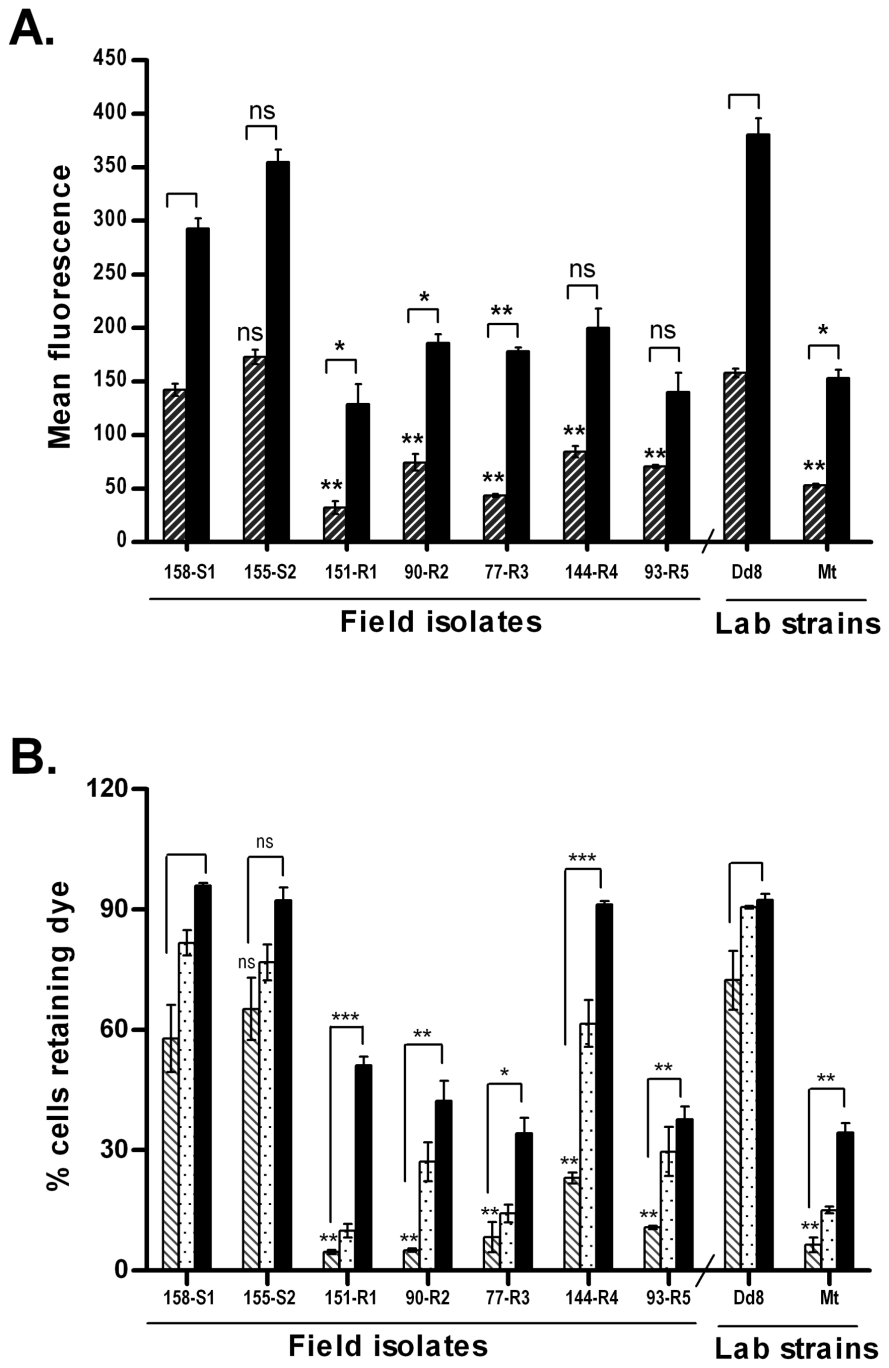
Effect of verapamil on the accumulation and retention of Rhodamine 123 in *L. donovani* isolates. A. Accumulation under normal conditions after 1h loading (hatched bar), accumulation in presence of verapamil (black bar). B. Retention of Rho 123 after 1 h of efflux (hatched bar), retention in presence of verapamil at the time of accumulation (white dotted bar), retention in presence of verapamil at the time of efflux (black bar). Results are mean of three independent experiments performed from three different promastigote cultures. * P ≤ 0.05, ** P ≤ 0.005, *** P ≤ 0.0005 indicate statistical significance with respect to reference sensitive isolate S1 for resistant isolates (R1- R5) and strain Dd8 for lab resistant mutant Mt, ns indicates no statistically significant difference.

Figure 2A depicts an accumulation of Rho123 in promastigotes in absence or presence of verapamil. In absence of verapamil, all the resistant isolates including resistant mutant Mt, exhibited significant lower accumulation of Rho 123 as compared to sensitive isolates S1, S2 and Dd8 (hatched bar). In the presence of 100 µM verapamil, there was a significant increase in the accumulation of Rho123 in all the isolates (black bar) irrespective of their sensitive or resistant nature. However, the fold increase in dye accumulation was significantly higher in all resistant isolates except R4 and R5 as compared to S1. Comparable fold increase in Rho accumulation was also observed in Mt as compared to Dd8. These observations suggest that verapamil is able to block the efflux of Rho 123.

To test whether the reduced accumulation in resistant cells is due to increased P-gp mediated efflux of the dye, the cells were preloaded with Rho 123 for 1h. After washing, the cells were transferred to dye free medium and percent cells positive for fluorescence (efflux) were measured. A significant decrease in percent dye positive cells were observed in all resistant isolates (Figure 2B, hatched bar) as compared to sensitive isolate S1. Sensitive isolates (S1, S2 and Dd8) exhibited >62% dye positive cells. Therefore, the percent cells that effluxed out the dye were only 36, 31.58 and 23.46%, in S1, S2 and Dd8, respectively. On the other hand, in case of resistant isolates, 76-95% cells had been effluxed out the dye (Figure 2B, hatched bar). Mt also exhibited 91.5% efflux.

Addition of verapamil at the time of dye accumulation inhibited Rho 123 efflux from both sensitive and resistant isolates (Figure 2B white dotted bar). However, this inhibition was more significant in resistant isolates as compared to sensitive ones. As compared to S1, maximum inhibition (50%) was observed in R4 followed by R2 and R5 (23 and 25 % respectively). Mt also showed blocking of efflux (17%) as compared to Dd8 strain. No significant inhibition was observed in R1 and R3. Interestingly, addition of verapamil at the time of dye efflux caused more significant inhibition of the dye efflux from cells as compared to its addition during accumulation. Sensitive isolates, S1, S2 and Dd8, in presence of verapamil at the time of efflux, exhibited more than 95% dye positive cells suggesting complete blockage of efflux (black solid bar). Resistant isolates also exhibited significant increase in percent dye positive cells. Here again, R4 exhibited a maximum increase in percentage dye positive cells from 23% to ^~^91%. The data indicates that the dye efflux is partially mediated through P-gp type MDR pumps and the blockage by verapamil is reversible in nature.

#### Trifluoperazine (TFP) blocked the efflux of Rho 123 irreversibly

**Figure 3:**
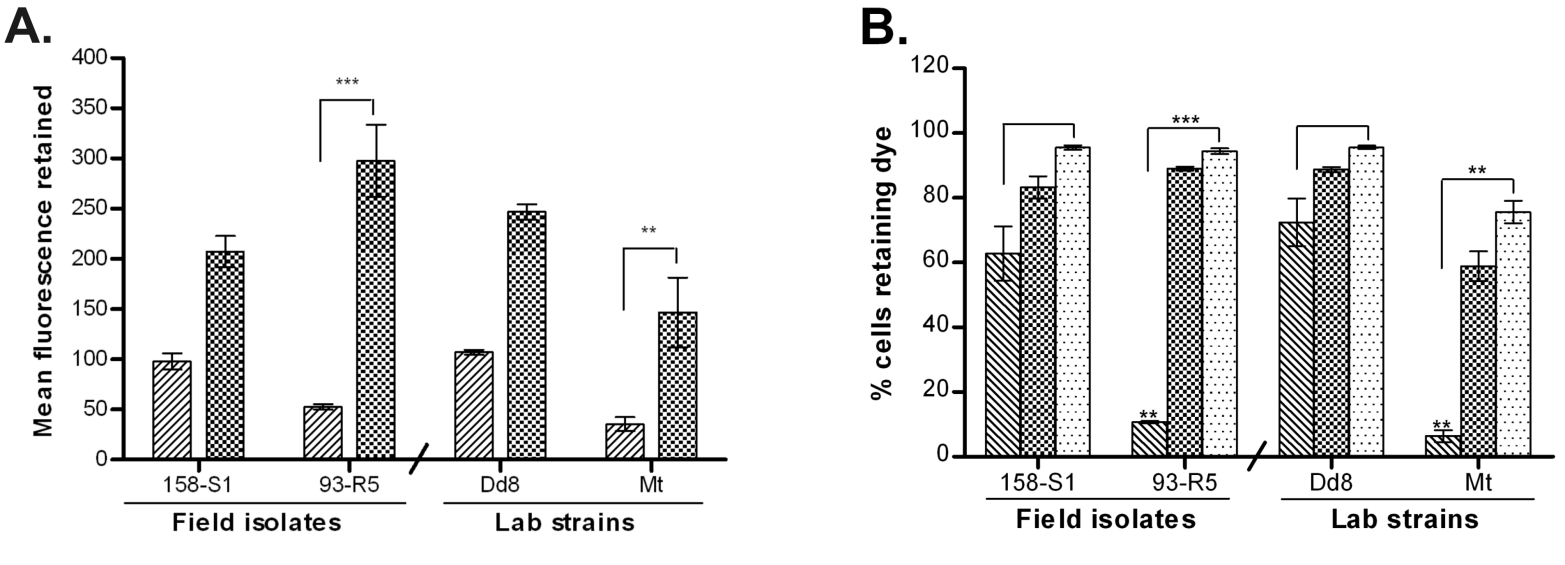
Effect of trifluoperazine on the transport properties of Rhodamine 123 in *L. donovani* isolates. A. Accumulation of Rho 123 after 1h loading (hatched bar1); accumulation in presence of TFP (mat bar). B. Retention of Rho 123 after 1 h of efflux (hatched bar), retention in presence of TFP at the time of accumulation (mat bar), retention in the presence of TFP at the time of efflux (white dotted bar). Results are mean of three independent experiments performed from three different promastigote cultures. * P ≤ 0.05, ** P ≤ 0.005, *** P ≤ 0.0005 indicate statistical significance with respect to sensitive isolate S1 for resistant isolates R5 and reference sensitive strain Dd8 for lab resistant Mt, ns indicates no statistically significant difference.

To confirm the involvement of P-gp mediated drug efflux in resistance, effect of another P-gp blocker, TFP was studied on four isolates namely Dd8: the sensitive strain, S1: the sensitive field isolate, R5: the resistant field isolate and Mt: laboratory resistant mutant. In the presence of 20 µM TFP (Figure 3A), though the accumulation of Rho123 was significantly increased in all the isolates (mat bar) but the fold increase was significantly higher in resistant isolates (3.2- fold in R5 and 4.1- fold in Mt) as compared to the sensitive isolates (2.11 fold in S1 and 2.3 fold in Dd8).

Figure 3B compares the efflux of Rho123 by resistant and sensitive isolates in presence TFP. The treatment of the cells with TFP at the time of accumulation drastically reduced the efflux which was more significant in the resistant isolates (79% & 63% inhibition in R5 and Mt respectively) than the sensitive isolates S1 (20.2%) and Dd8 (16.4%) (hatched bar verses mat bar). On the other hand, when the preloaded cells were treated with TFP at the time of efflux, the pumps were almost completely blocked in all the isolates and hence no efflux (Figure 3B, white dotted bar) was observed suggesting irreversible blocking of efflux pumps by TFP.

#### Transport properties of Calcein

The neutral dye calcein-AM is a nonfluorescent substrate for both the efflux pumps, P-gp and multidrug resistant protein A (MRPA), whereas its hydrolyzed fluorescent product, calcein is effluxed out only by MRPA. Calcein AM was used to load the parasites with calcein. Two field isolates, one sensitive (158-S1) and one resistant (93-R5) along with lab sensitive strain Dd8 and mutant Mt were studied in combination with two blockers, probenecid, the MRPA blocker and TFP, the P-gp blocker.

#### Probenecid had no effect on the transport of calcein

**Figure 4:**
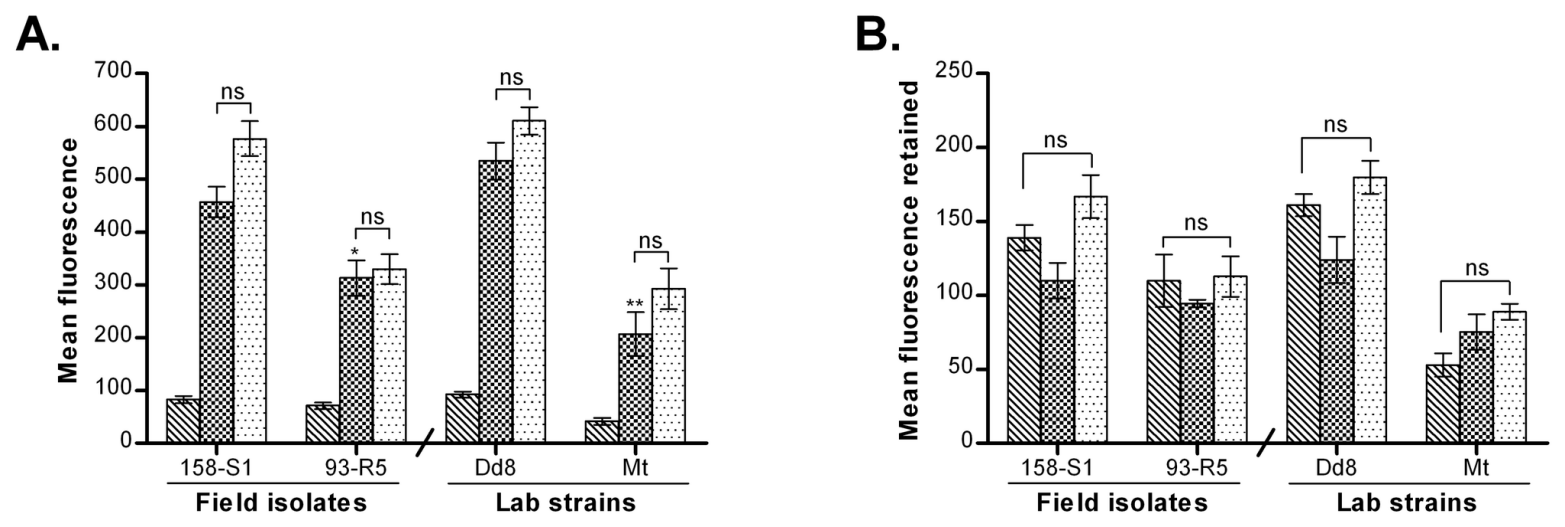
Effect of probenecid on accumulation and retention of calcein (Cal) in *L. donovani* isolates. A. Effect of probenecid on accumulation of Cal: accumulation under normal condition (hatched bar), accumulation under ATP depletion (mat bar), de-energized Cal accumulation in presence of probenecid (white dotted bar). B. Effect of probenecid on Cal efflux: Cal retention after 1 h 30 min efflux (hatched bar), retention of Cal in presence of probenecid at time of accumulation (mat bar), retention in presence of probenecid at time of efflux (white dotted bar). Results are mean of three independent experiments performed from three different promastigote cultures. * P ≤ 0.05, ** P ≤ 0.005 indicate statistical significance with respect to sensitive isolate S1 for resistant isolate R5 and reference sensitive strain Dd8 for lab resistant mutant Mt, ns indicates no statistically significant difference.

Figure 4A exhibits accumulation of calcein in the isolates. Under energized condition, very low fluorescence signal was detected (hatched bar) in all four cell types, which increased significantly under de-energized condition (after treatment with sodium azide) (Figure 4A, mat bar). This suggests that the efflux of calcein was very high under energized conditions in all the isolates hence exhibited very low accumulation of calcien. This increase in accumulation of calcein under de-energized conditions was significantly higher in sensitive isolates than in resistant isolates. Further, presence of probenecid did not have any significant effect on the accumulation of calcein in either of the isolates (Figure 4A, white dotted bar).

Interestingly, no significant difference was observed in the efflux of calcein by sensitive and resistant isolates (Figure 4B). The efflux of calcein was also not reversed by probenecid irrespective of the condition whether the blocker was added at the time of accumulation (mat bar) or efflux (white dotted bar).

#### Trifluoperazine blocked the efflux of calcein irreversibly

**Figure 5:**
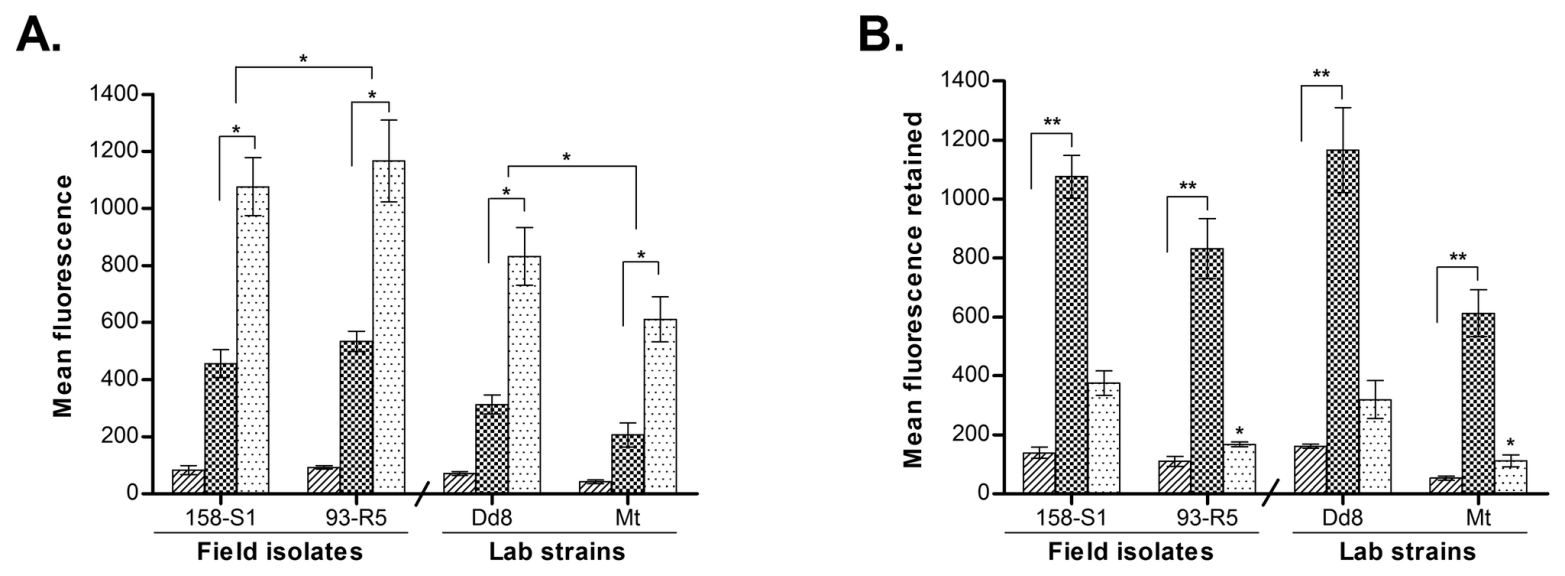
Effect of TFP on accumulation and retention of calcein (Cal) in *L. donovani* isolates. A. Accumulation of Cal: energized accumulation (hatched bar), de-energized accumulation (mat bar), de-energized accumulation in presence of TFP (white dotted bar). B. Effect of TFP on Cal efflux: Retention of Cal after 1 h 30 min (hatched bar), retention in presence of TFP at the time of accumulation (mat bar), retention in presence of TFP at the time of efflux (white dotted bar). Results are mean of three independent experiments performed from three different promastigote cultures. * P ≤ 0.05, ** P ≤ 0.005 indicate statistical significance with respect to sensitive isolate S1 for resistant isolate R5 and Dd8 for lab resistant mutant Mt, ns indicates no statistically significant difference.

As shown in Figure 5A, addition of TFP (white dotted bar) at the time of accumulation of dye resulted in increase in the retention of calcein in both sensitive and resistant isolates but the fold increase was higher in resistant isolates than in sensitive isolates.

Unlike Rho123, the efflux of calcein in absence of any inhibitor did not significantly differ in sensitive and resistant isolates (Figure 5B, hatched bars). Addition of TFP, at the time of accumulation, completely inhibited the efflux of calcein and the isolates retained all the calcein that was accumulated (mat bar). This efflux blocking by TFP was irreversible in nature as even after washing off TFP prior to efflux, the cells did not show any significant decrease in the mean fluorescence. On the other hand, addition of TFP to preloaded cells at the time of efflux, showed partial blocking of efflux pumps (white dotted bar). Sensitive isolate, S1 effluxed out 17.7% of accumulated calcein while 46.6% was effluxed by R5. Dd8 effluxed out 25% dye in presence of TFP whereas resistant mutant Mt effluxed 46.11% calcein (white dotted bar) as compared to untreated cells (hatched bar).

#### Intracellular thiol levels are elevated in SAG resistant isolates

**Figure 6:**
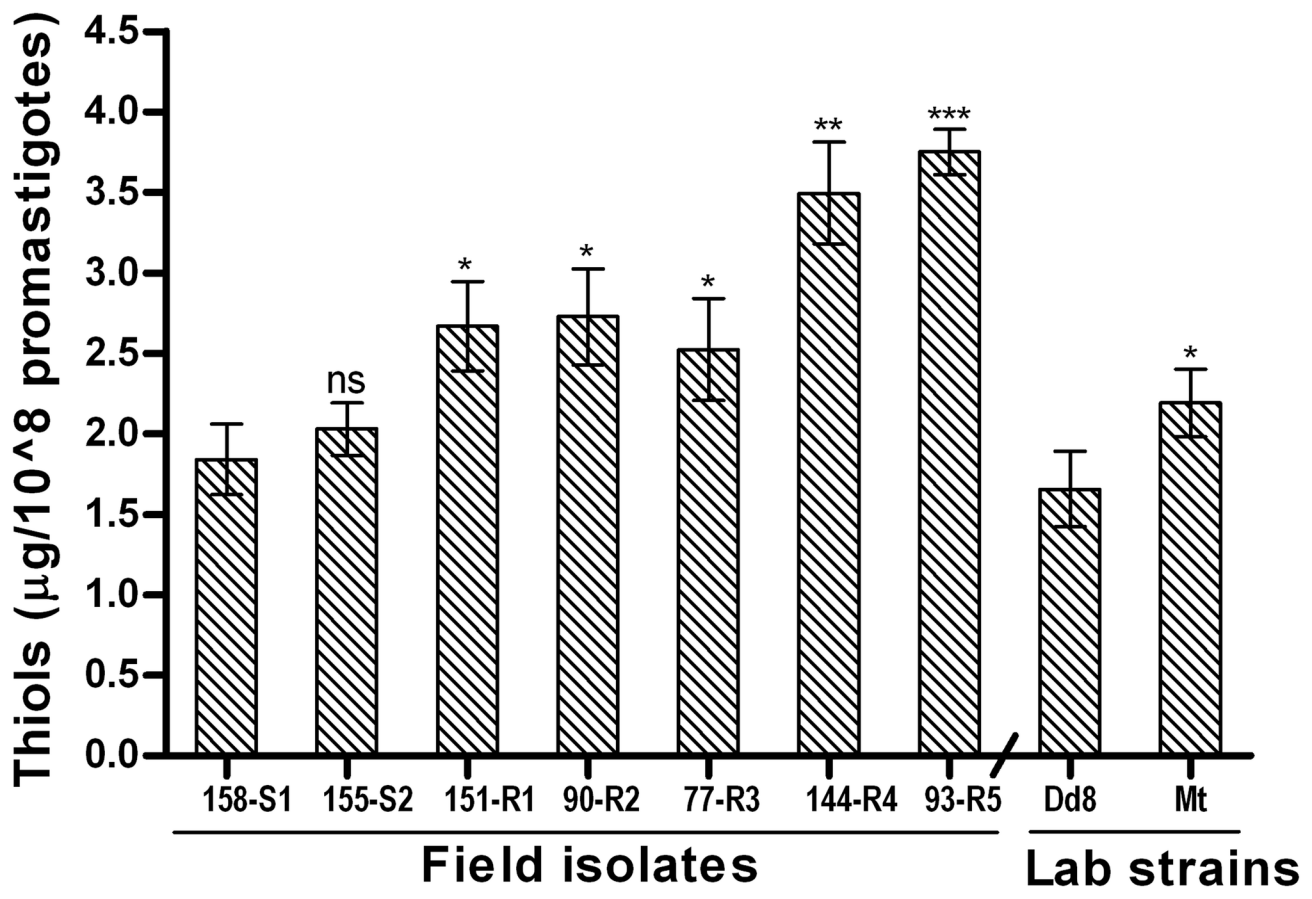
Total intracellular thiol levels in *L. donovani* isolates. Values are the mean ± SD of three experiments. * P ≤ 0.05, ** P ≤ 0.005, *** P ≤ 0.0005 indicate statistical significance with respect to sensitive isolate S1 for resistant isolates (R1- R5) and reference sensitive Dd8 for resistant mutant Mt, ns indicates no statistically significant difference.

Measurement of total intracellular non-protein thiol levels in *L. donovani* isolates indicated that all resistant isolates (R1-R5 and Mt) exhibited significantly higher levels of thiols than the three sensitive isolates (S1, S2 and Dd8) as shown in Figure 6. The increase in thiol levels in resistant isolates was 1.36 to 2.03-fold as compared to that of S1. Mutant Mt showed 1.38 fold increase in thiol levels as compared to Dd8. The thiol levels of sensitive isolates S2 and Dd8 (2.03 ± 0.16 μg/10^8^ and 1.65 ± 0.23 μg/10^8^ promatigotes) were comparable to that of S1 strain (1.84 + 0.22 μg/10^8^ promastigotes). To determine whether the observed increase in thiol levels was due to increase in trypanothione/its precursors or ovothiol too, the cells (both resistant and sensitive) were treated with 5mM BSO for 48h to deplete intracellular glutathione and related thiols and then allowed to regenerate thiols for 3h. Treatment with BSO resulted in significant decrease in both sensitive and resistant isolates (Table S1). However the decrease in resistant isolates was much more (7-10 fold) as compared to sensitive ones (5-6 fold). The presence of background fluorescence at ‘0’ min (after BSO treatment) possibly represents ovothiol [33] that cannot be inhibited by BSO [38]. Interestingly, this fluorescence was almost same in both resistant and sensitive strains.

#### Analysis of enzymes involved in thiol metabolism

Increase in thiol levels in resistant but not in sensitive isolates, suggests that resistant parasites modulate their thiol metabolism. This prompted us to study the expression pattern of three genes involved in the thiol metabolism. The proteins, ornithine decarboxylase (ODC) and gamma glutamyl cysteine synthetase (γ-GCS) are involved in thiol (glutathione and trypanothione) biosynthesis [39,40] whereas, trypanothione reductase (TR) helps in maintaining intracellular reducing environment [41].

#### γ-GCS is non-consistently up regulated

**Figure 7:**
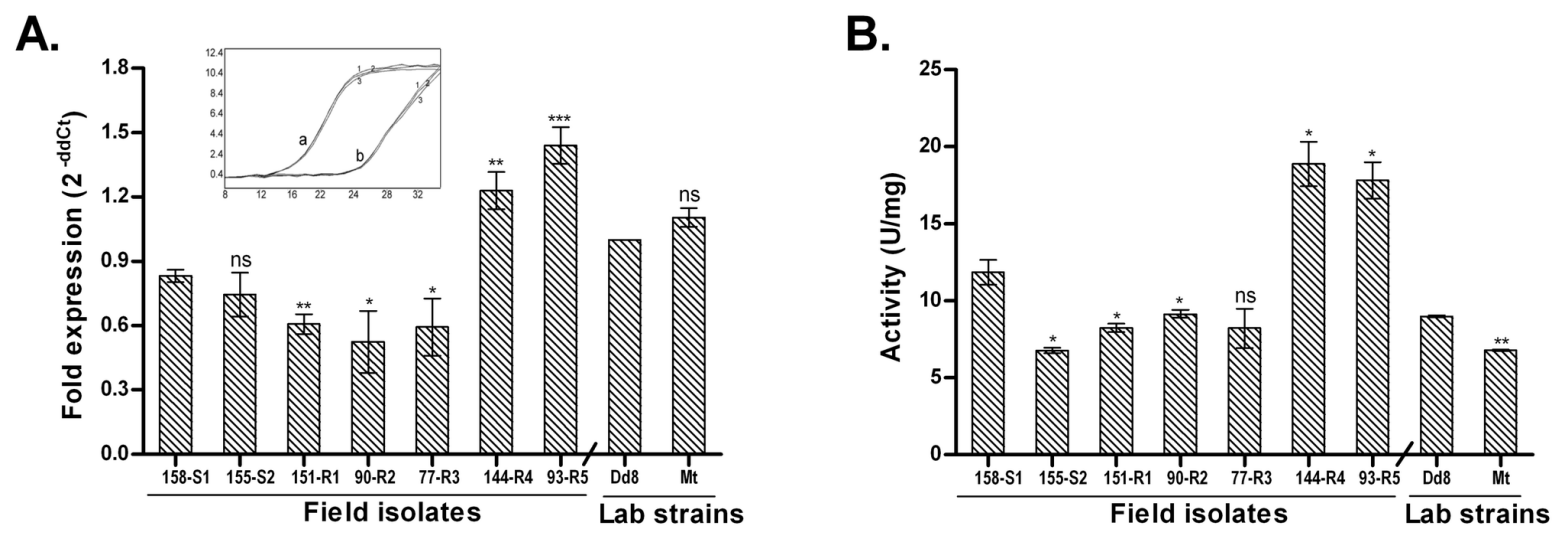
Expression analysis of γ-GCS in clinical isolates of *L. donovani*. A. Real time PCR expression ratios of resistant isolates (R1-R5) relative to sensitive isolate S1 and resistant mutant Mt relative to Dd8. B. Specific activity of γ-GCS. Results are mean of three independent experiments. * P ≤ 0.05, ** P ≤ 0.005, *** P ≤ 0.0005; indicate statistical significance with respect to sensitive strains, ns indicates no statistically significant difference. Inset shows the RT-PCR amplification curves; set a: curves for alpha tubulin amplification, set b: represents γ-GCS amplification (curve1: Dd8, 2: 144-R4, 3: Mt). X axis represents PCR cycle and Y-axis represents fluorescence.

Figure 7A depicts transcript levels of γ-GCS in field isolates. The sensitive field isolates S1, S2 and Dd8 showed comparable levels of γ-GCS expression. Among the resistant strains, R4 and R5 exhibited up regulation (1.47and 1.73 -fold respectively) whereas transcript levels of R1, R2 and R3 were down regulated in comparison to sensitive isolate S1. When compared to Dd8, the resistant mutant Mt displayed no significant change in transcript levels.

#### γ-GCS enzyme activity

To confirm the differential expression of γ-GCS in clinical resistance, enzymatic activity was determined in lysates of clinical isolates. Specificity of the assay was checked with γ-GCS inhibitor, L-buthionine-(SR)-sulfoximine (BSO) which inhibited the enzyme activity completely at 2.5 mM concentration as reported earlier [40]. As shown in Figure 7B, sensitive isolates S2 and Dd8 exhibited 42.8% and 24.2% decrease in the γ-GCS enzymatic activity respectively when compared to sensitive isolate S1. γ-GCS activities of resistant isolates R1 and R2 were significantly down-regulated whereas R4 and R5 possessed significantly up-regulated enzyme activity as compared to S1. Activity of R3 was comparable to S1. In comparison to reference sensitive strain Dd8, the γ-GCS activity of lab resistant mutant Mt exhibited a significantly lower enzymatic activity (24.5%). The enzymatic activities correlated well with the RNA levels of γ-GCS in isolates but not in Dd8 and Mt. No explanation can be offered for this observation at this point.

#### ODC is over expressed in resistant isolates

**Figure 8:**
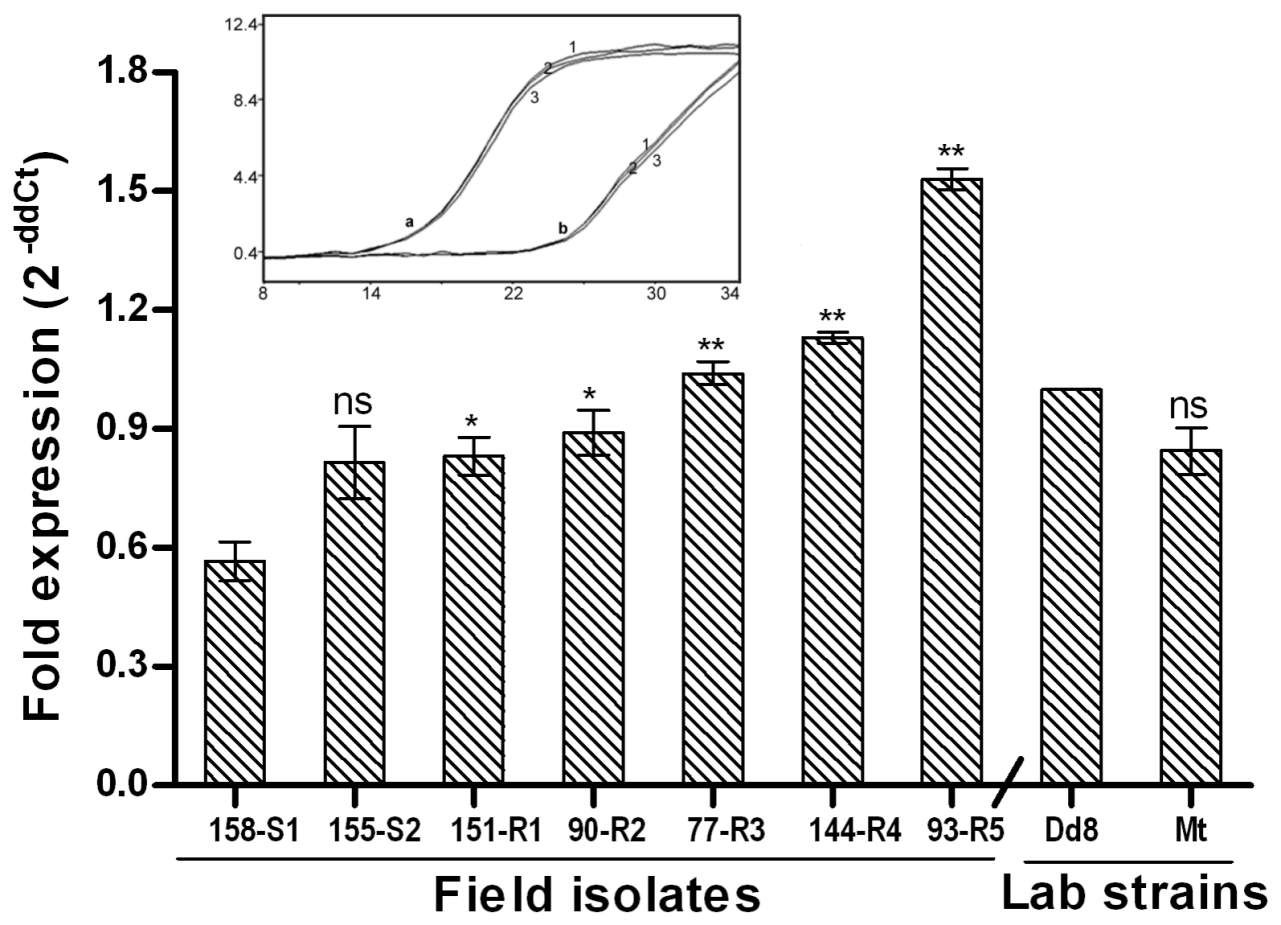
Real time PCR expression analysis of ornithine decarboxylase, one of target genes from the thiol metabolic pathway of *L. donovani* isolates. Expression ratios of resistant isolates (R1-R5) relative to sensitive isolate S1 and resistant mutant Mt relative to Dd8 Results are mean of three independent experiments performed from three different RNA preparations. * P ≤ 0.05, ** P ≤ 0.005, *** P ≤ 0.0005; indicate statistical significance with respect to reference sensitive strains; ns indicates no statistically significant difference Inset shows the RT-PCR amplification curves; set a: curves for alpha tubulin amplification, set b: curves for ODC amplifications respectively (curve1: Dd8, 2: 144-R4, 3: Mt). X axis represents PCR cycle and Y-axis represents fluorescence.

The transcript levels of ODC were comparable in the sensitive isolates S1 and S2 but sensitive strain Dd8 showed significantly increased (1.76-fold as compared to S1) transcript levels of ODC (Figure 8). All the resistant isolates possessed significantly increased transcript levels of ODC (1.46-fold to 2.7 –fold) as compared to S1. When compared to reference sensitive strain Dd8, **t**he transcript levels of resistant mutant Mt were unaltered.

#### Expression of TR

**Figure 9:**
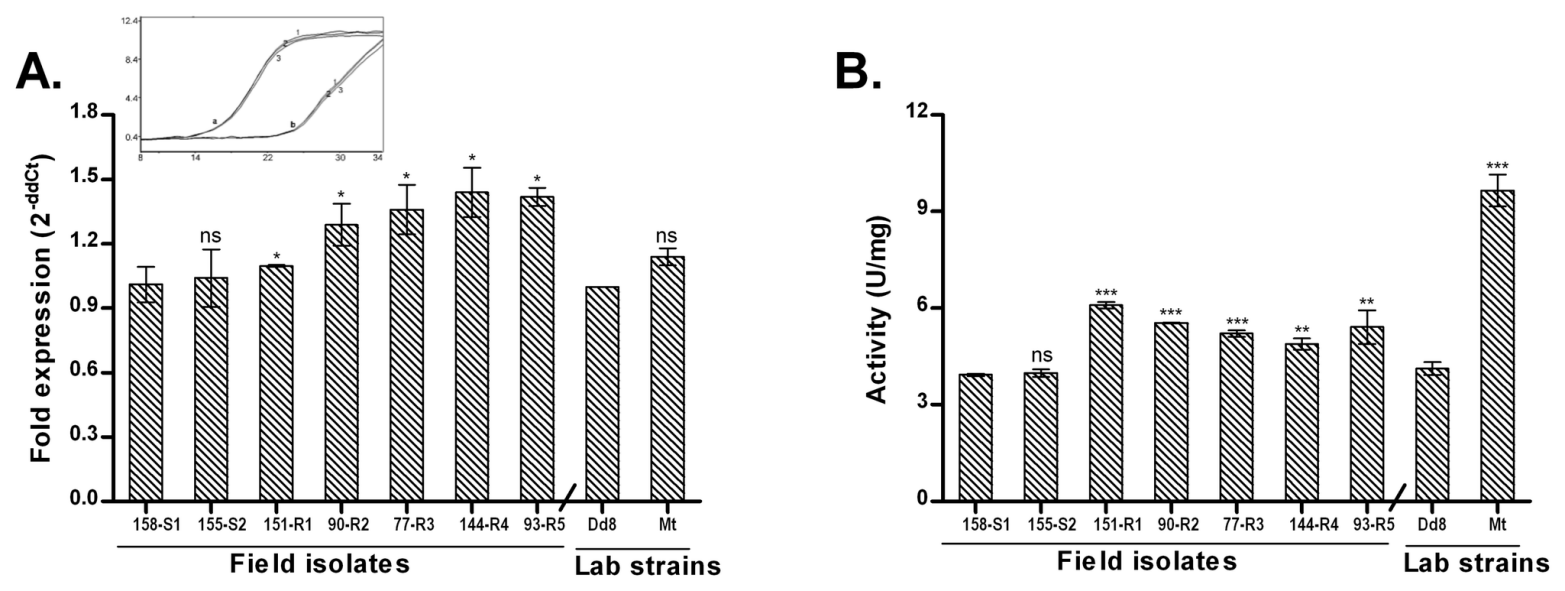
Expression analysis of trypanothione reductase in clinical isolates of *L. donovani*. A. Real time PCR expression ratios of resistant isolates (R1-R5) relative to sensitive isolate S1 and mutant Mt relative to Dd8. B. Specific activity of TR. Results are mean of three independent experiments. * P ≤ 0.05, ** P ≤ 0.005, *** P ≤ 0.0005; indicate statistical significance with respect to sensitive strains, ns indicates no statistically significant difference. Inset shows the RT-PCR amplification curves; set a: curves for alpha tubulin amplification, set b: represents TR amplification (curve1: Dd8, 2: 144-R4, 3: Mt). X axis represents PCR cycle and Y-axis represents fluorescence.

Transcript levels of TR were comparable among the sensitive isolates S1, S2 and Dd8 (Figure 9A). All resistant isolates exhibited significantly up-regulated transcript levels of TR as compared to S1. When compared to the lab sensitive strain Dd8, transcript levels of mutant Mt were unaltered.

#### Enzyme activity of TR

Similar to the expression analysis of TR, all resistant isolates displayed significantly increased TR enzymatic activities than the sensitive isolates (Figure 9B). In comparison to the activity of sensitive isolate S1, resistant isolates R1 to R5 displayed 1.24 to 1.55 fold increase in activity whereas fold increase for Mt was 2.34 fold when compared to Dd8.

## Discussion

In view of limited alternative treatments [10,5], and lack of effective vaccination [4], resistance to antimonials has emerged as the major pitfall in the treatment of Leishmaniasis. Major cause for drug resistance in various diseases, such as cancer, is the decreased intracellular concentration of drug or its active derivative either due to decreased uptake or increased efflux or a combination of both processes. In *Leishmania* , aquaglyceroporin1 (AQP1), member of the aquaporin superfamily has been shown to facilitate uptake of active form of antimonial drug, the trivalent antimony (SbIII) [13,14]. Over-expression of AQP1 in *L. major* (LmAQP1) produces hypersusceptibility to SbIII, whereas gene deletion renders the parasite resistant [14,42]. Previous studies on visceral clinical isolates from Nepal indicated that one of the mechanisms of antimony resistance was down-regulation of AQP1. However, similar data was not consistently observed in Indian isolates [19], suggesting that down regulation of AQP1 gene may not be a universal mechanism in all the visceral isolates. Interestingly the present study reports a significant down regulation of RNA transcripts of AQP1 in all the resistant isolates including laboratory resistant mutant. The down regulation is more significant in isolates with higher resistance indices (Figure 1A). This finding is in agreement with another report on Indian isolates by Mandal et al., 2010 [43]. Our data evidently substantiates that the down regulation of AQP1 is one of the resistance mechanisms in *L. donovani* isolates from the same geographical area (neighboring countries, India and Nepal).

Another resistance mechanism, responsible for lower concentration of drug in the cell is rapid efflux. The ATP-binding cassette (ABC) superfamily of proteins has been widely reported to export xenobiotics [44,45] outside the cell. These include the P-glycoprotein (P-gp) and multi-drug resistance- related protein (MRP). P-gp type efflux pumps play role mostly in resistance to hydrophobic compounds while MRP type pumps are known for efflux of anionic compounds in conjugation with thiols [45]. In *Leishmania* , several ABC transporters have been reported and characterized in relation to drug resistance. The first ABC transporter identified and characterized was MRPA. It was shown to confer antimony resistance by sequestering thiol-metal conjugates in an intracellular vesicle [15]. Other ABC proteins were also reported and linked to resistance but their exact mode of conferring resistance could not be ascertained. These included LtrABC1.1 [46] and LtrABCA2 [47] of ABCA subfamily, MDR1 of the ABCB subfamily [48-52] and also members of the ABCC subfamily [15,53,54]. Since all members of the ABCC subfamily are now shown to be localized intra-cellularly [55], it is clear that the efflux pumps on plasma membranes in *Leishmania* are unrelated to ABCC family. Further, role of ABCB proteins in antimony resistance and the nature of the thiol-X pump reported in *L* *. tarentolae* [55] are still not clear. Therefore, functional analysis of the efflux transporters in clinical isolates could provide an insight on the role of these proteins/efflux pumps in antimony resistance under natural conditions.

Sensitivity to vanadate [56] and ouabain resistance [57] are characteristic features of P-type ATPases. To date, increased P-ATPase activity has only been reported in methotrexate resistant *L. tropica* [29] and arsenite resistant *L. donovani* [58] laboratory mutants. Our results for the first time demonstrated the activity of the vanadate sensitive P-type ATPases in plasma membrane fractions of *L. donovani* field isolates (Table 3). The increased P-ATPase activities in resistant isolates suggests that efflux mediated antimony resistance may be operational in clinical isolates of *L. donovani*.

These findings were further corroborated by the functional assays of efflux pumps. The resistant isolates exhibited decreased accumulation and increased efflux of one of P-gp substrates, Rhodamine 123, than sensitive isolates. Further, the efflux of Rho123 was significantly inhibited in presence of verapamil, the pgp pump inhibitor (Figure 2A, B). This inhibition of efflux was partial and reversible. Though, our findings, are not in agreement to earlier studies [26,59] conducted on only one or two isolates but in agreement to more recent report [60]. It has been shown that verapamil sensitive pgp type pumps are expressed in high copy number in antimony resistant isolates of *L. donovani*. MDR1 gene has also been shown to be amplified in 65% of clinical isolates of *Leishmania* from Sudan and France [61]. Further, verapamil is also known to partially affect the non-MRPA thiol-X pump reported in *L* *. tarentolae* [55], therefore suggesting the pgp type nature of this efflux pump. Interestingly, the partial inhibitory effect of verapamil on P-gp pumps also provides an explanation for the reversal of resistance by verapamil in combination with SAG not only in *L. donovani* lab mutant [62] but also in clinical isolates [63]. The complete inhibition of Rho 123 efflux by trifluoperazine (TFP, another P-gp blocker), in both sensitive and resistant isolates (Figure 3B) further confirms the presence of P-gp type efflux pumps in *L. donovani* promastigotes. Taken together, for the first time, functionality of verapamil sensitive P-gp type efflux pumps was demonstrated in *L. donovani* antimony resistant field isolates.

Calcein AM (Cal-AM), another substrate for P-gp pump, freely permeates the cell membrane and is converted from a non-fluorescent substrate to fluorescent calcein (Cal) via intracellular nonspecific esterases, a substrate for MRP1/MRPA [64]. The probe calcein also registered lower accumulation in resistant isolates as compared to sensitive isolates (Figure 4). Further, Cal accumulation was not affected by the classical MRP blocker probenecid. The data suggests the absence of classical MRP pumps on parasite membrane which is also in accordance to earlier reports [26]. MRPA is reported to be expressed on membranes of intracellular vacuole of *Leishmania* to sequester drug-trypanothione conjugates inside the vacuole [15]. In contrast to probenecid, trifluoperazine blocked the Cal efflux activity completely and irreversibly (Figure 5A,B). Being P-gp inhibitor [64], TFP blocks the P-gp pumps and hence inhibits the efflux of P-gp substrate i.e. Cal AM, which is subsequently converted to Cal by cellular esterases and hence high levels of fluorescence of Cal were detected. An active efflux of Cal AM in promastigotes of *L. braziliensis, L. guyanensis and L. mexicana* had been reported earlier which was also accompanied by slow conversion of Cal AM to Cal [65]. In *L* *. brazileiences* , TFP mediated inhibition of Cal and Cal AM efflux was also reported [65] but the extent of pump blocking was different, which may be due to polymorphism in the efflux pumps among the species. Polymorphisms and point mutations in ABC drug efflux pumps have been identified in human populations which in few cases resulted in altered efflux properties [66,67]. In fact, bioinformatics analysis of ABC transporters of *Leishmania* spp revealed very low level of similarity even within strains of identical species that are prone to mutations [68], hence for resistance. TFP was reported to inhibit the efflux of pentamidine from *L. mexicana* resistant mutant [69] but had no effect on accumulation of pentamidine in *L. donovani* resistant mutant [70]. Therefore, it is evident that properties of membrane transporters in *Leishmania* appear to be species specific. The significant finding of our study is the complete and irreversible inhibition of efflux of all the three substrates i.e Rho 123, Cal and Cal AM by TFP. This implies that the efflux transporters of *L. donovani* isolates possess broad substrate specificity that may be helping parasite to evade therapeutic drugs in use by acquiring resistance against them.

The main characteristic of MDR efflux pumps is that the energy required for transport is derived from the activity of calcium-dependent ATPase. Since, phenothiazines inhibit the binding of calcium to calmodulin or calmodulin-type proteins [71] or acts as calmodulin antagonist [72] or calmodulin type proteins [73], hence, they were considered as potential inhibitors of MDR efflux pumps. Indeed phenothiazines have been shown to inhibit the efflux pumps that account for antibiotic resistance in cancer cells [74] and reverse antibiotic resistance of bacteria [75,76]. Our study has established TFP as an absolute inhibitor of plasma membrane efflux system in *L. donovani* clinical isolates from India. Role of MRPA in resistance in visceral isolates was confirmed by gene expression studies. All resistant field isolates of *L. donovani* as well as the lab mutant Mt exhibited up-regulation of MRPA (Figure 1B), which is in accordance to previous reports [25]. Interestingly, this up-regulation was also related to antimony resistance indices; higher the resistance index, higher was the fold up-regulation of MRPA. These results suggest that apart from efflux mechanism, *Leishmania* parasites adopt the strategy of drug sequestration in case of high resistance.

The role of elevated intracellular thiols in *in-vitro* as well as in clinical resistance is now well established [17,24,77,78]. Thiols have dual role in antimony resistance i.e., sensitization of the parasites by reducing SbV to SbIII [79-82] and promoting resistance by forming conjugates with SbIII for efflux and/or sequestration. Also higher levels of thiols in resistant isolates protect the parasites from Sb-mediated oxidative stress. In accordance to our previous report [24], we repeatedly observed 1.36 to 2.03 fold increase in total intracellular thiol levels in resistant isolates as compared to sensitive isolate S1 and 1.32 fold increase in mutant strain Mt when compared to lab sensitive Dd8. In *L. donovani* promastigotes, in addition to trypanothione, the major intracellular thiol, ovothiol is also present at significant levels (5%-35%) [83]. However, the observed increase in intracellular thiols in resistant isolates is due to increase in levels of glutathione related thiols. It is evident by comparable levels of non glutathione thiols, (Table S1) [33], observed in both sensitive and resistant cells after BSO treatment. After three hour regeneration, resistant cells exhibited again increased intracellular thiol levels as compared to sensitive ones. Therefore it is confirmed that up-regulation of intracellular non-protein thiols has emerged as a biomarker for clinical resistance in *L. donovani.*

In antimony-resistant laboratory mutants, increase in thiol levels is partially linked to amplification of γ-GCS [39] and ODC [40]. We found that γ-GCS exhibited increased RNA levels in highly resistant isolates (R4, R5) (Figure 7A) while in others, the expression was either unchanged or down regulated which is in accordance to earlier studies [20-22] including Indian isolates [25]. The mosaic pattern of γ-GCS expression at transcript levels was also confirmed at enzyme activity level (Figure 7B). Thus our earlier [24] and present work establishes that thiol up regulation in natural antimony resistance in *L. donovani* is not associated with γ-GCS up regulation as was observed for *L. mexicana* and *L. tropica* mutants [84].

In accordance to earlier reports [23,25], ODC exhibited an increased expression in *L. donovani* resistant isolates but not in lab mutant Mt (Figure 8). Interestingly, the ODC gene is present on chromosome 12, the chromosome reported to undergo aneuploidy (chromosome loss, partial haploid) in antimony resistant mutants of *L. infantum* [85]. Therefore, increased expression of ODC may be related to increased mRNA stability hence increased protein concentration [25]. Earlier SbIII resistant mutant of *L* *. tarentolae* also did not exhibit ODC up regulation [78] though it was up regulated in arsenite resistant mutants. Thus it appears that the expression status of ODC in clinical resistance is still open for study to establish its role in resistance.

Another pivotal enzyme of the thiol metabolism responsible for maintaining the intracellular reducing environment through trypanothione is TR. Our earlier work had highlighted the role of TR in natural antimony resistance [24]. Present study further confirmed increased RNA levels as well as enzyme activity of TR in resistant isolates as well as the mutant Mt (Figure 9). Expression rate of TR was also increased in SbV resistant clinical isolates of *L. braziliensis* [23]. Thus it can be concluded that up regulation of TR is an invariant feature of clinical resistance in *L. donovani* from India*.*

In conclusion, the present study for the first time established the role of P-gp like efflux transporters in clinical resistance in *L. donovani* and provided the functional evidence that MRPA pumps are not present on the parasite plasma membranes. Rather unique P-gp type pumps are suggested that transported the substrates of both P-gp and MRPA pumps and were completely inhibited by trifluoperazine. It was also confirmed that increased drug efflux, sequestration and reduced uptake are the main mechanisms adopted in high level of antimony resistance in field isolates of *L. donovani*. Further, levels of intracellular thiols are elevated, to aid the formation of drug thiol complexes to be effluxed out by membrane pumps or sequestration into vesicles. Indeed, thiol up regulation has emerged as an invariable feature of clinical resistance in *L. donovani* and can be proposed as a biomarker for clinical resistance. Thiol up-regulation in *L. donovani* is mediated by the increased expression of ODC and TR. γ-GCS plays role only in highly resistant isolates. In summary, for the first time, the role of plasma membrane efflux transporter(s) was demonstrated in antimony resistance in *L. donovani* field isolates. Further, decreased levels of AQP1 and elevated thiols levels have emerged as biomarkers for clinical resistance.

## Supporting Information

Table S1 **Total intracellular thiol levels in *L. donovani* promastigotes before and after BSO treatment.**
(DOCX)
