# Supplementary material for: Role of Efflux Pumps and Intracellular Thiols in Natural Antimony Resistant Isolates of Leishmania donovani
Source: PLoS One. 2013 Sep 17;8(9):e74862. doi: 10.1371/journal.pone.0074862 (PMC3775726; doi:10.1371/journal.pone.0074862)
Supplement: Table S1 — Total intracellular thiol levels in L. donovani promastigotes before and after BSO treatment. (DOCX) [file pone.0074862.s001.docx]

**Table S1**. Total intracellular thiol levels in *L. donovani* promastigotes before and after BSO treatment.

| **Total Intracellular Thiols**  (Mean Fluorescence Value) | | | | | |
| --- | --- | --- | --- | --- | --- |
| Strain | Before BSO treatment | After 5.0 mM BSO treatment^#^ | |  | |
|  |  | 0 min | 180 min | |  |
| **Dd8** | 644.27±41.24 | 134.42±15.48 | 194.23±33.37 | | |
| **Mt** | 842.56±64.81^*^ | 135.27±31.44^ns^ | 392.77±36.95^*^ | | |
| **158-S1** | 701.38±31.5 | 123.54±25.44 | 270.22±55.04 | | |
| **93-R5** | 1446.46±71.56^**^ | 144.87±22.38^ns^ | 419.03±52.07^*^ | | |

Values are the mean ± SD of three experiments Welch's correction was used to calculate mean significant diiference (*p≤0.05; ** P≤0.005; ns indicates no statistically significant difference) with respect to lab sensitive Dd8 for resistant mutant Mt and sensitive isolate S1 for resistant isolate R5

# Cells were suspended in BSO free fresh M199 medium supplemented with 10% Fetal calf serum and allowed to regenerate thiols for ‘0’ min and ‘180’ min.
